# The evolution of imprinting: chromosomal mapping of orthologues of mammalian imprinted domains in monotreme and marsupial mammals

**DOI:** 10.1186/1471-2148-7-157

**Published:** 2007-09-06

**Authors:** Carol A Edwards, Willem Rens, Oliver Clarke, Andrew J Mungall, Timothy Hore, Jennifer A Marshall Graves, Ian Dunham, Anne C Ferguson-Smith, Malcolm A Ferguson-Smith

**Affiliations:** 1 Department of Physiology, Development and Neuroscience, University of Cambridge, Downing Street, Cambridge CB2 3DY, UK; 2 Cambridge Resource Centre for Comparative Genomics, Department of Veterinary Medicine, University of Cambridge, Cambridge CB3 OES, UK; 3 Wellcome Trust Sanger Institute, Wellcome Trust Genome Campus, Hinxton, Cambridge CB10 1SA, UK; 4 Research School of Biological Sciences, The Australian National University, Canberra, Australia

## Abstract

**Background:**

The evolution of genomic imprinting, the parental-origin specific expression of genes, is the subject of much debate. There are several theories to account for how the mechanism evolved including the hypothesis that it was driven by the evolution of X-inactivation, or that it arose from an ancestrally imprinted chromosome.

**Results:**

Here we demonstrate that mammalian orthologues of imprinted genes are dispersed amongst autosomes in both monotreme and marsupial karyotypes.

**Conclusion:**

These data, along with the similar distribution seen in birds, suggest that imprinted genes were not located on an ancestrally imprinted chromosome or associated with a sex chromosome. Our results suggest imprinting evolution was a stepwise, adaptive process, with each gene/cluster independently becoming imprinted as the need arose.

## Background

Genomic imprinting is an epigenetic phenomenon that has been well-characterised in eutherian mammals. Imprinted genes are expressed from one of the two parentally inherited chromosome homologues and repressed on the other. The mechanism of parental-origin specific gene expression is associated with heritable differential modifications to the DNA and chromatin that are programmed during gametogenesis [1]. Since the discovery of imprinting in placental mammals over 20 years ago there has been much speculation about how the mechanism has evolved. Despite this, the range of mammalian species tested for imprinting is limited and very few non-mammalian vertebrates have been experimentally assessed. Mammals that diverged early from the lineage of eutherian mammals are ideally suited for investigating imprinting evolution by comparing epigenetic mechanisms within mammalian species. Such comparative analysis has wider implications for our understanding of the evolution of the epigenetic control of genome function. To date, based on investigations of eutherian imprinted orthologues, imprinting has been demonstrated at some loci in marsupials (both *Macropus eugenii *{tammar wallaby} and *Monodelphis domestica *{grey short-tailed opossum} which diverged from each other approximately 70 million years ago) but not in monotremes (platypus and echidna) [2-5]. This suggests that, if imprinting arose only once in mammals, it evolved somewhere between the divergence of monotremes (prototherians) from therian mammals around 166 million years ago (MYA) [6] and the divergence of marsupials (metatherians) from eutherian mammals approximately 147 MYA.

The egg-laying monotreme is an important link between birds and viviparous mammals, and is therefore of interest for studies on the evolution of imprinting. In addition, the platypus has been shown to possess 10 sex chromosomes, 5 Xs and 5 Ys [7,8]. In male meiosis these 10 chromosomes form a multivalent chain consisting of alternating X and Y chromosomes [7]. The 5Y and 5X chromosomes segregate alternately from a translocation chain to form male (5Y) and female (5X) determining sperm. Dosage compensation mechanisms have not been elucidated in monotremes. Parallels have been drawn between epigenetic mechanisms associated with genomic imprinting and X chromosome dosage compensation in female eutherian mammals. Hence determining the presence, organisation and location of imprinted orthologues in the monotreme can provide a useful framework for comparative mechanistic and evolutionary studies.

Recently, different views on the evolution of imprinting mechanisms have been expressed. Two views are based on the similarities between X chromosome inactivation (XCI) and autosomal genomic imprinting that have long been noted [9]. Since both have a number of features in common, such as the association with non-coding and anti-sense RNA and some related patterns of histone modifications, it has been suggested that X-inactivation was the 'driving force' behind the evolution of imprinting [10]. This idea has grown from the finding that, in marsupials, XCI is an imprinted event with the paternal X being preferentially inactivated in all tissues [11,12]. In *Mus musculus *(mouse) and *Bos taurus *(cow), imprinted XCI is an early event confined to extra-embryonic tissues [13,14] and occurring prior to the reprogramming of the X in the epiblast which leads to random XCI in embryonic derivatives [15,16]. Once inactivation was fixed on the X chromosome in ancestral mammals, it has been suggested that these mechanisms were adopted by autosomes to establish genomic imprinting[10]. An alternative to the 'driving force' hypothesis is the view that imprinting and X-inactivation co-evolved when the placenta emerged [17]. In this perspective, the evolution of placentation exerted selective pressure to imprint growth-related genes present on both the X and the autosomes. The basis of this model is the suggestion that genes imprinted in the placenta utilise a non-coding RNA mechanism that parallels the function of the Xist non-coding RNA essential for X inactivation in placental mammals. Most recently, data have emerged proving that marsupial imprinted X-inactivation and platypus sex chromosome dosage compensation occur via a mechanism that is independent of the *XIST*-mediated mechanism occurring in mouse and man [18,19]. This finding is not consistent with either of the two proposed models linking X inactivation to autosomal imprinting.

Another theory postulates that imprinted domains evolved through chromosomal duplication and that imprinted genes were originally located on one (or a few) ancestral pre-imprinted chromosome region(s) and then dispersed in mammalian genomes through recombination or transposition events [20]. Duplication of a set of genes may have led to random monoallelic expression as a means of dosage compensation and, subsequently, imprinting (parental-origin specific gene activity/repression) following divergence of the paralogues. If imprinted genes were found to be located on one or two platypus autosomes this would constitute some evidence for this hypothesis. Alternatively, given the large number of platypus sex chromosomes that may have epigenetically regulated dosage compensation mechanisms, it is possible that autosomal imprinted domains might have arisen through translocation of sex chromosome-linked genes onto autosomes carrying with them vestiges of the regulatory sequences required for parental origin specific sex chromosome dosage compensation. It is relevant to note however, that the platypus sex chromosome system bears no relationship to the XY system in viviparous mammals (Rens et al. submitted for publication).

In order to understand the emergence of imprinting after the divergence of monotremes from the mammalian lineage we have isolated platypus (*Ornithorhynchus anatinus*) and tammar wallaby (*Macropus eugenii*) bacterial artificial chromosome (BAC) clones that contain orthologues of mouse and human imprinted domains and investigated their localisation on tammar wallaby and platypus chromosomes. We have determined the chromosomal location of 8 imprinted gene orthologues in the platypus, representing 7 different clusters of imprinted genes in the mouse or human (the *IGF2 *imprinted domain, *IGF2R*, the *DLK1/DIO3 *imprinted domain, *GRB10*, the *GNAS *complex, a gene from the Prader-Willi/Angelman Syndrome complex and *SLC38A4*). In addition 8 imprinted gene orthologues were mapped in the tammar wallaby – a ninth was mapped previously. Three of these genes belong to the Beckwith-Wiedemann Syndrome (BWS) orthologous region and two to the *DLK1/DIO3 *region. The genes investigated here represent the best-characterised imprinted domains known in the mammalian genome and can be considered in the context of the information available on their imprinting status. Our analysis contributes to the identification of regions of syntenic homology across a range of vertebrates including chicken and the prototherian, metatherian and eutherian mammals.

## Results

### Identification of platypus and tammar wallaby BACs containing imprinting orthologues

**Figure 1:**
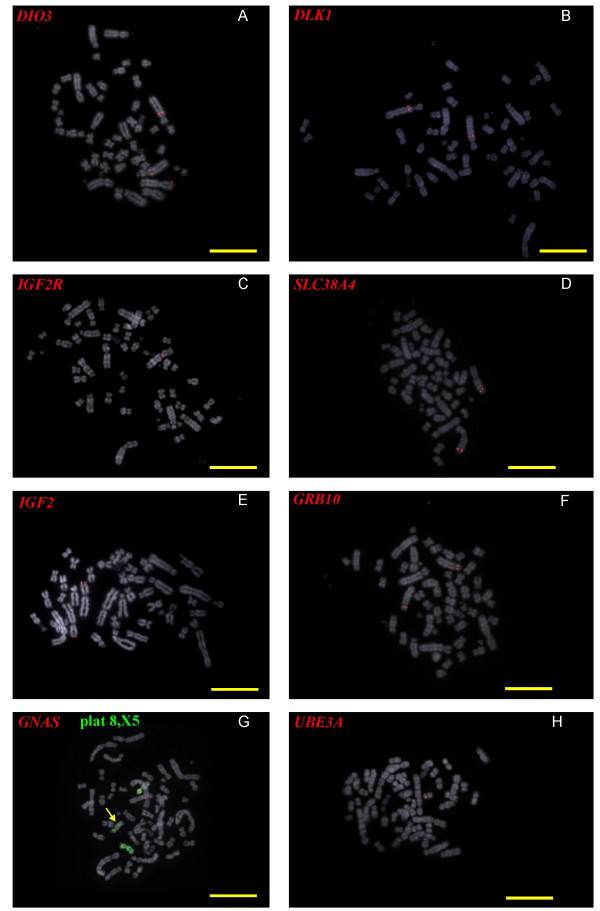
**FISH mapping on platypus metaphase chromosomes of BACs containing orthologues of imprinted genes**. **(A) ***DIO3*, **(B) ***DLK1*, **(C) ***IGF2R*, **(D) ***SLC38A4*, **(E) ***IGF2*, **(F) ***GRB10*, **(G) ***GNAS *(and platypus 8 paint in green) and **(H) ***UBE3A*. Scale bar is 10 μm.

**Figure 2:**
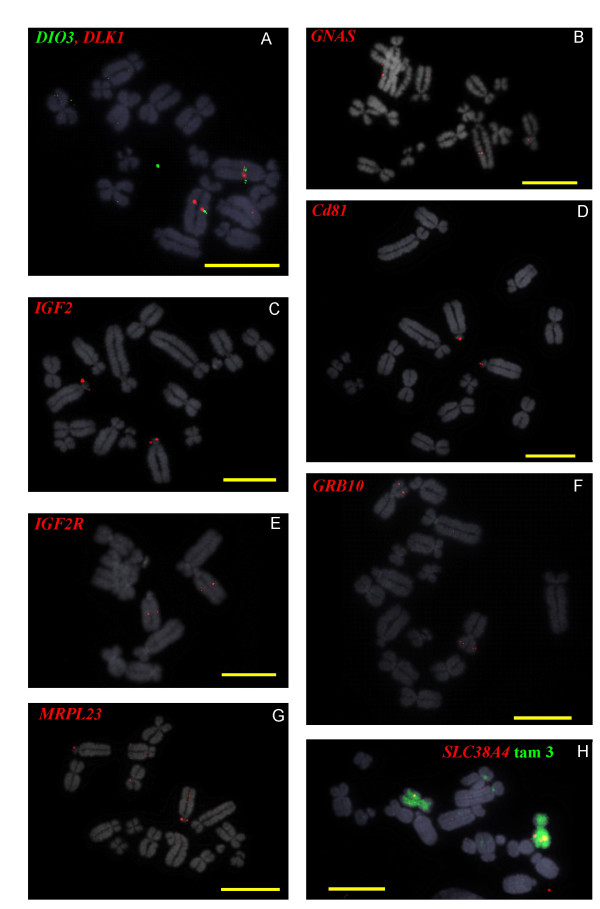
**FISH mapping on tammar metaphase chromosomes of BACs containing orthologues of imprinted genes**. **(A) ***DIO3 *(green) and *DLK1 *(red), **(B) ***GNAS*, **(C) ***IGF2*, **(D) ***CD81*, **(E) ***IGF2R*, **(F) ***GRB10*, **(G) ***MRPL23*, and **(H) ***SLC38A4 *(red) with chromosome 3 in green. Scale bar is 10 μm.

Each of the imprinted genes described in this report have been mapped by fluorescent in situ hybridisation (FISH) of BAC clones to metaphase chromosomes of platypus and wallaby cells in culture to determine their regional position and in some instances, to confirm retention of clustering across the cluster (Figures 1 &2).

The orthologues of both the insulin like growth factor 2 (*IGF2*) and one of its receptors (*M6P/IGF2R*), have previously been characterised in the platypus [Genbank:AY552324 and Genbank:AF151172] [3,21]. *IGF2 *is a paternally expressed imprinted gene in both eutherian and marsupial mammals but has been shown not to be imprinted in birds and monotremes [2,4,22]. In mouse and human it forms part of a large imprinted cluster that can be divided into two imprinted subdomains – one containing the *IGF2 *and *H19 *genes, and the other containing *CDKN1C *and several genes showing tissue-specific imprinting in the mouse placenta including *CD81*. These two contiguous subdomains map to chromosome 11p15.5 in humans (BWS critical region) and mouse distal chromosome 7. A fragment of *IGF2 *was amplified from platypus DNA using primers from the highly conserved second coding exon C2 in platypus. This was used as a probe to screen platypus and wallaby BAC libraries. *M6P/IGF2R *is a large gene consisting of 48 exons which encodes a protein of 2482 amino acid residues in mouse. It is expressed from the maternally inherited chromosome in mice [23] and has also been shown to be imprinted in the opossum *Didelphis virginiana*[3]. This gene is biallelically expressed in monotremes and also lacks IGF2 binding properties in these species [3]. To screen the wallaby BAC library, a probe was designed to *Macropus rufogriseus *(red-necked wallaby) *IGF2R *mRNA [Genbank:AF339159].

The other genes/regions chosen for this study had not previously been characterised in monotremes or marsupials. The *CD81 *gene encodes a member of the transmembrane 4 superfamily which is preferentially expressed from the maternal allele in mouse placentas [24]. *CD81 *is approximately 240 kb downstream of *IGF2 *in human. A probe of the entire human *CD81 *coding sequence was used to screen the wallaby BACs and 5 positives were found. *DIO3 *is an intronless gene that codes for type III iodothyronine deiodinase (D3), a 278 amino acid selenoprotein in human. It is a predominantly paternally expressed gene which is part of the *DLK1/DIO3 *cluster which is found at 14q32 in humans and distal chromosome 12 in mice. DLK1 is a Delta-like protein member of the Notch family of signalling molecules and is found in all vertebrates. Despite this *DLK1 *is not as conserved as the other imprinted genes in this study so in order to produce probes to screen the libraries, the trace archives from NCBI were searched with *DLK1 *sequences from other species. By searching the *Monodelphis domestica *trace archive with human *DLK1 *[Genbank:NM_003836], TI_395847291 was identified and a probe designed to the most conserved regions between the two sequences was used to screen the wallaby library. Chicken *DLK1 *sequence [Genbank:XM_421369] identified the platypus trace file TI_752207707 to which a probe was designed to screen the platypus library. The growth factor receptor-bound protein 10 gene (*GRB10*) is expressed from the paternally inherited chromosome in both mouse and human brain. In other organs, it is maternally expressed in mouse and biallelically expressed in the human. It appears to be a solitary imprinted gene which is located on human 7p12 and mouse proximal 11. The GNAS complex is located on human 20q13.3 and mouse distal 2. This is a complex domain with a number of differentially imprinted, alternatively spliced transcripts. The guanine nucleotide binding protein, alpha stimulating gene (*GNAS*) is highly conserved in vertebrates. The Prader-Willi/Angelman Syndrome cluster is located at human 15q11–13 and mouse central chromosome 7. This is a large cluster that spans 4 Mb in human and includes the ubiquitin protein ligase E3A gene (*UBE3A*) that is expressed from the maternally inherited chromosome. This gene has previously been assigned to wallaby chromosome 5 [25,26]. Finally, solute carrier family 38, member 4 (*SLC38A4 *also called *ATA3*) is located on human 12q13 and mouse distal chromosome 15. It is found in a gene cluster with two other solute carriers of which it is the only imprinted one, being repressed on the maternally inherited chromosome. Probes were designed to highly conserved regions in each of these genes and used to screen platypus and tammar wallaby BAC libraries.

Further information on all probes used for library screens, the sequences they were designed against and the number of BACs identified can be found in [see Additional file 1].

### FISH mapping of Platypus BACs

**Figure 3:**
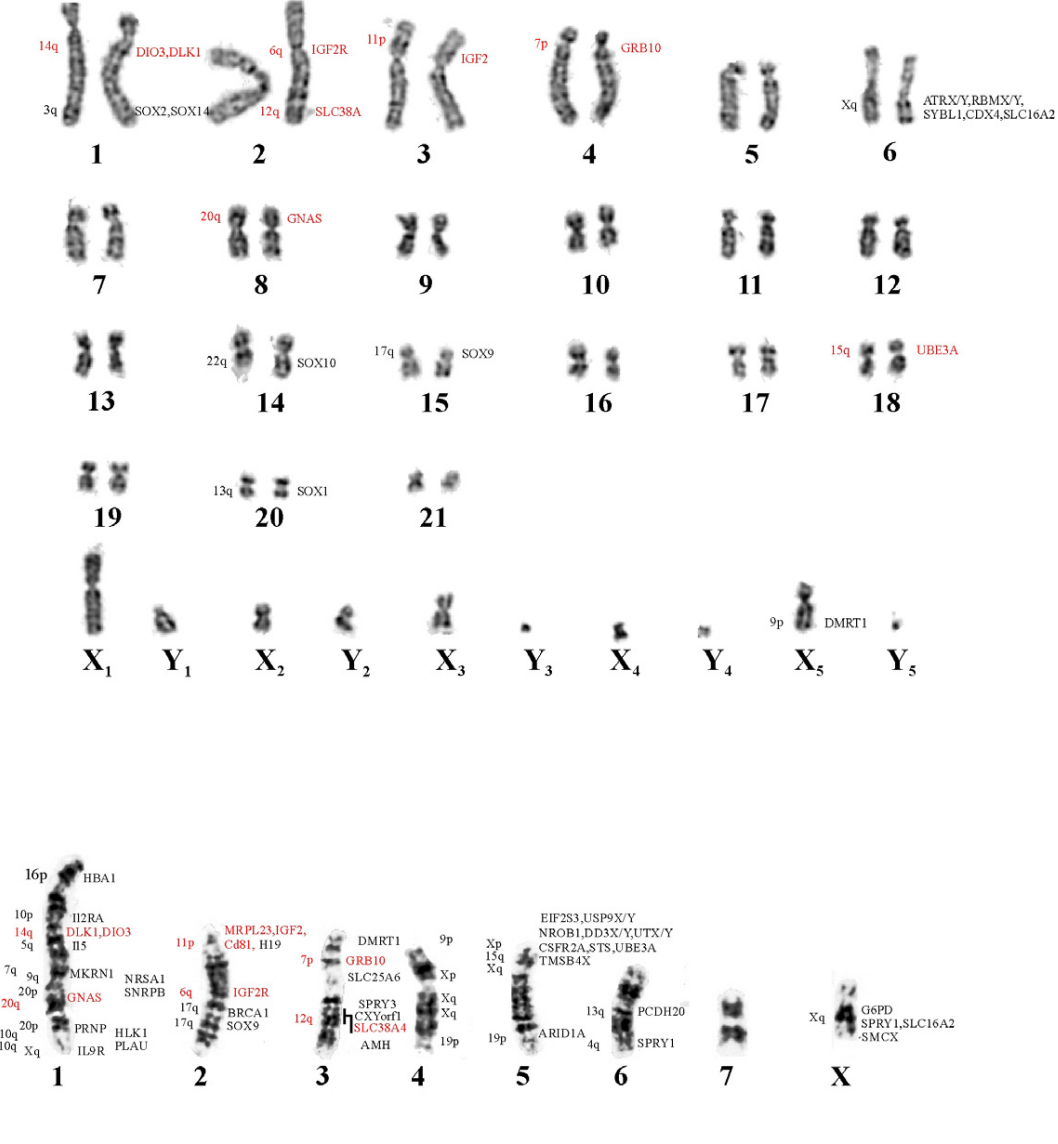
Location of orthologues of mammalian imprinted genes on the karyotypes of platypus **(A) **and tammar wallaby **(B) **in red. Gene names in black are those previously mapped genes from other studies, (reviewed in [25, 44]). The position of the orthologous genes in human are shown on the left.

The platypus karyotype (2 n = 52) consists of 21 autosomes and 10 sex chromosomes (5X's and 5Y's in male and 5 X-pairs in female). One positive BAC for each gene was chosen for FISH analysis. The BACs were labelled with biotin using a standard nick translation protocol and localised on platypus chromosomes by FISH on male platypus metaphase preparations. Fig 1a shows the localization of *DIO3 *to a site distal to the centromere of platypus chromosome 1. *DLK1 *maps close to *DIO3 *in platypus (Fig 1b) *IGF2R *and *SLC38A4 *both localise to platypus chromosome 2, *IGF2R *to a position close to the centromere of chromosome 2, and *SLC38A4 *to distal 2q (Fig 1c and 1d). *IGF2 *maps to distal platypus chromosome 3p (Fig 1e). *GRB10 *is positioned near the centromere of platypus 4 (Fig 1f). Fig 1g shows *GNAS *on platypus chromosome 8 as confirmed by FISH using a chromosome 8 specific paint. A fainter signal was also observed on platypus X_5_. *UBE3A *is found on platypus chromosome 18 (Fig 1h). All gene locations are shown on the platypus G-banded karyotype (Fig 3).

### FISH mapping of Tammar Wallaby BACs

The tammar wallaby karyotype (2 n = 16) consists of 7 autosomes and the two sex chromosomes. The tiny Y chromosome is not shown in Figure 3. The genes were localised on male tammar wallaby metaphase chromosomes using FISH with labelled BAC DNA (as above). *DIO3 *and *DLK1 *(Fig 2a) were mapped to tammar wallaby chromosome 1q about one third distal from the centromere. *GNAS *also was mapped to chromosome 1q but considerably more distal from the centromere (Fig 2b). *IGF2, CD81, and MRLP23 *were mapped to the same cytogenetic region on tammar wallaby chromosome 2p (Fig 2c, 2d and 2g). *GRB10 *localised to tammar wallaby 3p (Fig 2f).*IGF2R *was mapped to 2q, half way down that arm (Fig 2e). *SLC38A4 *was mapped to tammar wallaby chromosome 3p, as confirmed by chromosome painting with a chromosome 3 specific paint (Fig. 2h).

### Conservation of synteny

*Dlk1 *and *Dio3 *encompass a 1 MB region in the mouse. In order to ascertain whether synteny is conserved within the *DLK1*/*DIO3 *domain, *DLK1 *containing BACs were identified in both species. One BAC from each species was used for FISH analysis. *DIO3 *(Fig 1a) and *DLK1 *(Fig 1b) mapped to a site on the long arm 1/3 the arm length from the centromere of platypus chromosome 1. In tammar wallaby *DLK1 *and *DIO3 *also mapped to the same location as shown by FISH analysis with the probes labelled in two different colours (Fig 2a).

Lambda clones containing *IGF2 *have previously been mapped to tammar wallaby chromosome 2p [27]. In order to confirm this location and see if synteny was conserved in this species, BACs containing 2 other genes from this region were isolated. *CD81 *is preferentially expressed from the maternally inherited allele in mouse placentas. *MRPL23 *is located 175 kb upstream of *IGF2 *in humans and it encodes the mitochondrial ribosomal protein L23. This gene does not appear to be imprinted in mammals. Hence the genes selected here fall into three different functional and regulatory categories which may not have conserved ancestral linkage. For example the two different imprinted subdomains might be separated from each other and/or the unrelated mitochondrial protein. One positive BAC for each of these genes was used for FISH which showed that *IGF2*,*CD81 *and *MRPL23 *do indeed map together on tammar wallaby chromosome 2p (Fig 2c, 2d and 2g).

The location of imprinted orthologues in the chicken by *in-silico *methods has been recently published [28]. We have also performed an *in-silico *analysis to identify the chromosomal locations of the imprinted genes in the opossum using the UCSC genome browser[29,30]. The results of this analysis and the FISH mapping are summarised in Table 1.

**Table 1:** Summary of chromosomal locations of genes studied in human, mouse, wallaby, opossum, platypus and chicken

**Gene**	**Human location**	**Mouse location**	**Wallaby location**	**Opossum location**	**Platypus location**	**Chicken location**
DIO3	14q	Distal 12	1q	1	1q	5
DLK1	14q	Distal 12	1q	1	1q	5
GNAS	20q	Distal 2	1q	1	8p	20
GRB10	7p	Proximal 11	3p	6	4p	2
IGF2	11p	Distal 7	2p	5q [35]	3p	5
CD81	11p	Distal 7	2p	Unplaced	-	5
MRPL23	11p	Distal 7	2p	Unplaced	-	5
IGF2R	6q	Proximal 17	2q	2	Centric 2	3
SLC38A4	12q	Distal 15	3q	8	2q	1
UBE3A	15q	Central 7	5 [23]	7	18p	1
SNRPB	20p	2	1q [23]	1	-	20

Chicken and opossum locations are taken from the UCSC genome browser except for opossum *IGF2 *[35]

### *In silico *identification of orthologues and chromosomal locations of genes contiguous with imprinted genes

The transcripts of the human imprinted genes were aligned by BLAST to find orthologues within the Ensembl Platypus *Ornithorhynchus anatinus *database release 5. The platypus contigs in the database contain several predicted genes, which were then identified by blasting to find alignments with the NCBI human genome database. Orthologues of these genes were subsequently localised in chicken and opossum by BLAST alignment in Ensembl. The results are shown in Table 1 and Table 2.

**Table 2:** *In silico *identification of orthologues and chromosomal locations of genes contiguous with imprinted genes.

**Platypus Contig**	**Predicted gene**	**Human orthologue**	**Human location**	**Chicken assignment**	**Opossum assignment**
1q Ultracontig378	Ox_plat42681	LGMN	14q32.12	5	1
	Ox_plat497848	High E-value	-	-	-
	Nov499641/Nov5433	GOLGA5	14q32.12	5	1
	Nov5434	CHGA	14q32.12	5	1
	Ox_plat52244	ITPK1	14q32.12	5	1
	Ox_plat7773	MOAP1	14q32.13	5	-
	Ox_plat1058	C14orf130	14q32.13	5	1
	Nov0444/Ox_plat88666	BTBD7	14q32.13	5	1
	Ox-plat454053/Nov11241/Ox_plat35285	KIAA1409	14q32.13	5	1
	Ox_plat548259	High E-value	-	-	-
	Nov11242/Ox_plat367667	ASB2	14q32.13	5	1
	Nov11243	High E-value	14q32.13	-	-
	Nov11244/Ox_plat4560	OTUB2	14q32.13	5	1
	Nov11245	KIAA1622	14q32.13	5	1
	Ox_plat116474	KIAA1622	14q32.13	5	1 insertion
	Nov11246/Ox_plat42680	High E-value	-	-	- insertion
	Nov12040/Ox_plat411980	SERPINA11	14q32.13	5	- insertion
	Nov12038/Ox_plat468381/Nov12037	High E-value	-	-	- insertion
	Nov12036	GSC	14q32.13	5	1 insertion
	Nov12034/Ox_plat475598	DICER1	14q32.13	5	1
	Nov12033	CLMN	14q32.13	5	-
	Nov9154	C14orf49	14q32.13	5	1
	Ox_plat409095	High E-value	-	-	-
	Nov65921/Nov6591	BDKRB2	14q32.2	5	1
	Nov65905/Ox_plat42182/Nov6589;6588	C14orf103	14q32.2	5	1
	Nov6588	C14orf129	14q32.2	5	1
	Nov13812/Ox_plat6298	PAPOLA	14q32.2	5	-
	Nov12141/Ox_plat6576	VRK1	14q32.2	5	1
	Nov14770	BCL11B	14q32.2	5	1
	Nov14773	KRT19	17q21.2	27	2
	Nov14774/Nov3153/Ox_plat403179	SETD3	14q32.2	5	1
	Nov2410	KIAA1822	14q32.2	5	1
	Nov7692	CYP46A1	14q32.2	5	-
	Nov7691	LOC91461	14q32.2	5	-
	Nov4045/Ox_plat43367	EML1	14q32.2	5	1
	Nov12424/Ox_plat123106	DEGS2	14q32.2	5	1
	Nov5723/Ox_plat43391	YY1	14q32.2	5	1
	Nov5724	SLC25A29	14q32.2	5	1
	Nov5726	C14orf68	14q32.2	5	1
	Nov5727/Ox_plat494845	WARS	14q32.2	5	1
	Nov5730	High E-value	-	-	-
*	Nov5731	DLK1	14q32.2	5	1
	Nov5732/Ox_plat43477	DNAH1	3p21.1	12	6
	Nov5733/Ox_plat522828	DYNC1H1	14q32.32	5	1
	Nov5734/Ox_plat6302	HSP90AA1	14q32.32	3	-
	Nov5735/Ox_plat461283	WDR20	14q32.32	5	1
	Nov5736/Ox_plat549051/Ox_plat5738/Ox_plat456837	RAGE	14q32.32	5	1
	Nov5740/Ox_plat526165	KIAA0329	14q32.32	5	1
	Nov5741	ANKRD9	14q32.32	5	1
	Nov9609	KIAA1446	14q32.2	5	-
8p Contig16	Ox_plat6649	CYP24A	20q13.2	20	1
	Ox_plat6759	PFDN	20q13.2	20	-
	Ox_plat44306	DOK	20q13.2	20	-
	Ox_plat1864	CBLN4	20q13.31	20	1
	Ox_plat373291	High E-value	-	-	-
	Ox_plat6760	CSTF1	20q13.31	20	1
	Ox_plat485472	C20orf32	20q13.31	20	1
	Ox_plat509072	C20orf43	20q13.31	20	1
	Ox_plat21038/Ox_plat49662	High E-value	-	-	-
	Ox_plat50570	BMP7	20q13.31	20	1
	Ox_plat6664	SPO11	20q13.32	20	1
	Ox_plat6762	RAE1	20q13.32	20	1
	Ox_plat501210	RBM30	20q13.32	20	-
	Ox_plat21169	CTCFL	20q13.32	20	1
	Ox_plat364767	PCK1	20q13.32	20	1
	Ox_plat50577	TMEPAI	20q13.32	20	1
	Nov 9262	TMEPAI	20q13.32	20	1
	Ox_plat21326	C20orf80	20q13.32	20	-
	Ox_plat21104	RAB22A	20q13.32	20	1
	Ox_plat21317	C20orf80	20q13.32	20	-
	Ox_plat69044	PPP4R1L	20q13.32	20	1
	Ox_plat50567	VAPB	20q13.32	20	1
	Ox_plat499610	STX16	20q13.32	20	-
	Nov9272	Fam38A	16q24.3	11	1
	Ox_plat499488	C20orf45	20q13.32	20	1
*	Nov9275	GNAS	20q13.32	20	1
	Ox_plat6777	TUBB1	20q13.32	-	1
	Ox_plat21080	High E-value	20q13.32	-	-
	Nov9282	C20orf174	20q13.32	20	1
	Ox_plat4456	PHACTR3	20q13.33	20	1
	Ox_plat454388	CDH26	20q13.33	-	1
4p Contig107	Ox_plat400863	CUL1	7q36.1	2	8
	Ox_plat10731	EZH2	7q36.1	2	3
	Nov0280	PDIA4	7q36.1	2	8
	Nov0281	High E-value	-	-	-
	Ox_plat49897	COBL	7p12.1	2	6
*	Ox_plat451754/Nov0283	GRB10	7p12.1	2	6
	Nov0284	DDC	7p12.2	2	6
	Nov0285	FIGNL1	7p12.2	-	6
	Ox_plat403769/Nov0286	IKZF1	7p12.2	2	6
	Nov0287	ZPBP	7p12.2	2	6
	Nov0288	High E-value	-	-	-
	Ox_plat4844817/Ox_plat467096	High E-value	-	-	-
2 Contig1301	Nov7295	SLC22A2	6q25.3	3	2
*	Q9N1T1	IGF2R	6q25.3	3	2
2q Contig538	Nov6345/Nov6346/Ox_plat486528	SLC38A1	12q13.11	1	8
*	Nov6348	SLC38A4	12q13.11	1	8
18p Contig121	Ox_plat15397	UBE3A	15q11.2	1	7
	Ox_plat498667	MGC26733	2q11.2	1	-
	Ox_plat3315	TMEM131	2q11.2	1	7
	Ox_plat59493	TMEM47	Xp21.2	1	4
	Nov7105/Nov7106/Ox_plat390388	High E-value	-	1	
	Ox_plat85743	CXorf22	Xp21.2	1	4
	Ox_plat472396	PRRG1	Xp21.2	1	4
	Ox_plat1731	XK	Xp11.4	1	4
	Nov7111	CYBB	Xp11.4	1	4
	Ox_plat85753	DYNLT3	Xp11.4	1	4
	Ox_plat514733	SYTL5	Xp11.4	1	4
	Ox_plat7251	SRPX	Xp11.4	1	4
	Ox_plat375200	RPGR	Xp11.4	1	4
	Ox_plat1440	OTC	Xp11.4	1	4

*DLK1 *is located on platypus 1q in ultracontig378 which contains 49 predicted genes, most of them with orthologues on human 14q, chicken 5, and opossum 1. The three genes that have orthologues elsewhere might be mistakes in the ultracontig assembly. Genes that are present on either side of *DIO3 *on human chromosome 14q are mapped in the same platypus ultracontig378. On opossum chromosome 1 *DLK1 *and *DIO3 *are 1.6 Mb apart according to Ensembl-opossum. The platypus ultracontig378 does not correspond to a continuous region in opossum. The predicted genes between *KIAA1622 *and *GSC *are not identified in opossum but instead are replaced by regions homologous to regions other then human 14q13.2 and chicken 5.

*GNAS *is located on platypus 8p in contig16 together with 31 other genes (4 unidentified) all of which have orthologues on human 20q13, chicken 20, and opossum 1. Only one gene (*Fam38A*) is located on human 16q and chicken 11 and is probably a mistake in this contig assembly. *GRB10 *was found on platypus 4p in contig107, which contains 13 other genes. All of the genes have orthologues on chicken 2. Three of them have orthologues on human 7q36.1 and the other eight are on human 7p12 (4 genes are unidentified). An inversion in the eutherian lineage separated these genes from each other. In the marsupial *Monodelphis domestica *these two gene clusters are not syntenic but are localized on different chromosomes (chromosome 8, 6 and 3 respectively, Ensembl Opossum release 4). *IGFR2 *and *SLC38A4 *are found in small contigs with a limited number of genes.

*UBE3A *is located on platypus 18p in contig121 together with 15 other genes (3 unidentified). All of the genes have orthologues on chicken 1. However, *UBE3A *is localized on human 15q. Two other genes are on human 2q and the remaining genes in this contig are on human Xp21.2 or Xp11.4. As these genes are syntenic in platypus and chicken, this contig represents the ancestral configuration. Before the marsupial-eutherian split, one fission separated the human Xp region from the human 15q and human 2q regions; the latter two regions are still together in opossum. A subsequent fission in the eutherian lineage separated the human 15q and human 2q regions. Unfortunately, *IGF2*, *CD81*, *MRPL23 *and *SNRPB *are not yet recognised in the Ensembl Platypus *Ornithorhynchus anatinus *database 5.

This approach identified conserved synteny at the majority of extended loci examined. We identified one large inversion, and potential errors in the platypus contig assembly. Finally, we determined that the *UBE3A *region on platypus chromosome 18 and chicken chromosome 1 represent an ancestral configuration of 15 genes which during eutherian evolution has undergone fission placing several of them on two regions on the human X chromosome.

## Discussion

Studies that consider the chromosomal relationships between autosomal imprinting and dosage compensation mechanisms in the range of mammals that include monotremes, marsupials, mouse and man are likely to provide insights into the evolution of the mechanisms involved. In a wider context, this will aid in understanding the evolution of epigenetic controls regulating genome function.

Monotremes, due to their early offshoot from the other mammalian species, are an ideal class for various kinds of genetic, cytogenetic and epigenetic research. Whereas most male mammals have an XY complement and female birds have a ZW complement, the male platypus has five X- and five Y chromosomes. Furthermore, X_5 _carries the *DMRT1 *orthologue present on the avian Z and thought to be sex determining. Platypus X_1 _was previously thought to show homology with the human X (see for example ref 29), but this is not confirmed by the draft platypus sequence (Ensembl release 44) that instead shows homology to chicken chromosome 3, 11, 12, 13, and Z and human chromosome 2 and 5 (Rens et al submitted). Therian X-linked genes mapped to date are predominantly localised to platypus chromosome 6 [31]. The results indicate that the monotreme sex chromosome system is unrelated to the XY sex chromosome system of other mammals which must have arisen after the divergence of monotremes 166 MYA. This intriguing system combined with an apparent absence of genomic imprinting makes it important to localize imprinted genes on platypus chromosomes in order to consider the evolution of epigenetically regulated dosage compensation systems. These localizations also serve to define regions of syntenic homology between vertebrates including monotremes and eutherian mammals. In addition, the placement of such genes on the cytogenetic map will contribute to anchoring the platypus genomic sequence currently being generated.

Here we mapped the chromosomal location of imprinted genes in the platypus and tammar wallaby. Eight imprinted gene orthologues (representing six different imprinted mouse/human clusters) localized to 6 different autosomes in the platypus as shown in Fig. 1a–h. In tammar wallaby eight imprinted gene orthologues (three belonging to the BWS region) representing the same six imprinted domains were mapped to 5 of the 7 different autosomes. First, the results will be discussed in relation to other genes mapped in platypus and tammar wallaby. Second, the imprinted gene orthologue localization will be discussed in relation to imprinting evolution.

### Comparative gene mapping

Gene mapping is one of the tools used to define regions that are conserved between different species. The localization of orthologues of imprinted genes (red) on platypus chromosomes is presented in Figure 3a with genes mapped previously in black [31-34]. Gene mapping data are still limited in platypus, hence mapping the orthologues of imprinted genes will anchor contigs to specific chromosomes and aid in constructing a platypus-human homology map.

The localization of orthologues of imprinted regions in tammar wallaby is presented in Fig 3b. We show that *IGF2 *is located at the telomere of tammar chromosome 2. A recent paper placed the *M. domestica *orthologue of *IGF2 *on 5q [35] a region that was previously shown to be equivalent to 6p in tammar [36]. This discrepancy might be due to the poor resolution of chromosome paints at the telomeres and suggests that there may be a small region at the tip of *M. domestica *5q which is homologous to 2p in the tammar wallaby. It is interesting that *GNAS *and *SNRPB *are close on tammar wallaby 1p (our results and Rapkins *et al*[26]), which is part of a region that is conserved in a large set of marsupial species[37]. *GNAS *is located on human distal 20p and *SNRPB *on distal 20q. Human chromosome 20 is a chromosome that is conserved in all eutherian mammals, the mapping of *GNAS *and *SNRPB *indicates that it is conserved in marsupials as well. The four homologous regions mapped in this report add to the complexity of the rearrangements that have occurred during chromosome evolution between human and tammar wallaby. For instance, tammar wallaby chromosome 1 has regions homologous to human 5, 7, 9, 10, 14, 16, 20 and X (our results and Alsop *et al*.[25])

### Imprinted gene orthologue localization

The overall conclusion made from the mapping data is that the orthologues of these imprinted genes are not found on sex chromosomes in either species. Although the mechanism of dosage compensation remains to be determined in platypus, the lack of imprinted orthologues on sex chromosomes does not favour the idea that imprinted genes arose as duplications from the X.

This, and the absence of imprinting in the platypus to date, suggests that monotreme X chromosome dosage compensation preceded genomic imprinting which subsequently adopted the same mechanism, or that sex chromosomes dosage compensation in monotremes is an unrelated event. The latter is more likely since monotreme sex chromosomes share no homology with the human X (Rens et al submitted). The position of orthologues of imprinted genes provides no insight regarding the hypothesis of co-evolution of X-inactivation and imprinting in mammals being associated with placentation [17].

The results show that the selected imprinted gene clusters are scattered among autosomes in the platypus and tammar wallaby karyotypes; the clusters do not group together in either species. Data from comparison of the distribution of the imprinted gene orthologues in platypus and tammar wallaby with their locations in the human karyotype reflects the high number of rearrangements that occurred in the lineages of either the monotremes or placental mammals. The position of genes on the prototherian ancestor will be more relevant to evaluating the imprinting duplication hypothesis and comparing it with data generated here. However, the prototherian ancestral karyotype remains to be determined and will be assisted by the establishment of a genome wide comparison between monotremes/marsupials and an outgroup species.

The *SNRPN *gene in the PWS/AS cluster arose from a tandem duplication of the *SNRPB *gene so its syntenic relationship with imprinted *GNAS *is of interest. The *SNRPB *duplication had already occurred when the marsupials diverged from the eutherian line as *SNRPB *and *SNRPN *are tandemly arranged in both tammar and opossum. *In silico *analysis of this region in the chicken shows that there is only one copy of *SNRPB *and that it is only 166.9 kb away from *GNAS *on chromosome 20 implying that these genes were close in the ancestral mammalian karyotype.

*In-silico *analysis reveals that *SNRPB *and *GNAS *are 36.6 Mb apart in the opossum and 54.5 Mb apart in human. Therefore although these two genes are located on the same chromosome they have become separated by one or more inversions. Furthermore, in opossum, tammar, platypus, chicken and zebrafish, the PWS/AS genes *SNRPN *and *UBE3A *are on separate chromosomes and are expressed biallelically in tammar [26]. Together these findings suggest that imprinted regulation was acquired after the loss of close synteny with *GNAS *and a major rearrangement that united *SNRPN *and *UBE3A*. However, it also remains theoretically possible that the *SNRPN *and *UBE3A *genes lost imprinting in the macropodid lineage and their imprinting state is ancestral for therians.

## Conclusion

The combined data of chicken, marsupial and platypus gene position suggest that imprinted gene orthologues have existed on separate chromosomes since before imprinting evolved. This makes the hypothesis, that there was a single or small number of ancestrally imprinted chromosomes, unlikely. The observation that some imprinted domains in mouse and human are not imprinted in marsupials, suggests that imprinting was a step wise process during evolution beginning after the evolution of viviparity and continuing convergently in the marsupial and eutherian lineages. Thus the evolution of imprinting has most likely been a long process with each cluster independently evolving or indeed losing, its imprinting mechanisms as the need arose. This suggests an element of adaptation in the process of imprinting evolution.

## Methods

### Amplification and sequence analysis

The published coding sequences of the genes of interest were obtained from as many species as possible from Entrez Gene at the NCBI webpage [38] [Additional File 1]. Sequences were then aligned to each other using the ClustalW program [39,40] and PCR Primers designed to the regions of greatest homology within the same exon.

Platypus genomic DNA (gDNA) was extracted from primary fibroblasts using standard protocols [41]. *IGF2*, *DIO3 *and *SLC38A4 *were amplified in a 15 μl reaction containing 1× NEB buffer [42], 500 μM dNTPs, 2.5 μg BSA (Sigma), 0.067% v/v β-mercaptoethanol, 0.6 U Taq polymerase (Applied Biosystems), 0.75 μM of each primer and 50 ng gDNA. *GNAS*, *GRB10 *and *IGF2R *were amplified in a 25 μl reaction containing 1× PCR Buffer (Bioline), 1.5 mM MgCl, 250 μM dNTPs, 1.5 U Taq polymerase (Bioline), 0.3 μM of each primer and 50 ng gDNA. PCR cycling was, 94°C for 5 min, 35 cycles at 94°C for 30 sec, annealing temperature (specific for each primer see table 1) for 30 sec, 72°C for 30 sec and 5 min at 72°C. *UBE3A *was amplified in a 25 μl reaction containing 1× PCR Buffer (KOD Hot Start, Novagen), 300 μM dNTPs, 1 mM MgSO_4_, 0.5 U Hot Start KOD polymerase, 0.6 μM of each primer and 50 ng gDNA. PCR cycling was 94°C for 2 min, 31 cycles of 94°C for 15 sec, 60°C for 30 sec, 68°C for 30 sec, then 5 minute at 68°C.

The PCR products were separated by electrophoresis and the appropriately sized fragments were excised and cleaned (Qiaquick Gel Extraction Kit; Qiagen). These fragments were cloned into pCR^® ^2.1-TOPO^® ^(Invitrogen) using the manufacturers protocol. DNA from the plasmids was prepared using GeneElute™ Plasmid Miniprep Kit (Sigma) then sequenced to confirm its identity.

### BAC Isolation

The OA_Bb Platypus BAC library (Clemson University Genomics Institute, South Carolina, USA) and the ME _KBa Tammar wallaby BAC library (Arizona Genomics Institute, USA) were screened with [α-^32^P] dCTP (Amersham Pharmacia Biotech) labelled PCR products. Labelling was performed under the following conditions 94°C for 5 minutes, 25 cycles of 93°C for 30 sec, 50°C for 30 sec 72°C for 30 sec and 1 cycle of 72°C for 5 min. Probes were denatured at 99°C for 5 min and snap chilled before hybridisation. The library membranes were hybridised and washed at low stringency (55°C). They were then exposed to X-ray film at -70°C overnight. BACs were streaked to single colony and tested by PCR with their identifying primers to ensure they contained the correct gene.

### Preparation of BAC Probes

BAC DNA was isolated using the protocol described at the Wellcome Trust Sanger Institute methods website [42]. The DNA probes were labelled by nick translation with Biotin-16-dUTP using a standard protocol.

### Localization of DNA probes

Chromosome specific DNA was prepared from flow-sorted platypus chromosomes and fluorescence in situ hybridization was performed according to protocols described previously [7,43]. The labelled DNA probes (and chromosome paints for chromosome identification) were hybridized to male platypus and wallaby chromosome preparations and detected with Cy3-avidin.

### Image analysis

Images were captured using the Leica QFISH software (Leica Microsystems) and a cooled CCD camera (Photometrics Sensys) mounted on a Leica DMRXA microscope equipped with an automated filter wheel, DAPI, FITC, and Cy3 specific filter sets and a 63×, 1.3 NA objective or 100×, 1.4 NA objective.

## Abbreviations

MYA – Million Years Ago

XCI – X Chromosome Inactivation

BAC – Bacterial Artificial Chromosome

FISH – Fluorescent *In-situ *Hybridisation

## Authors' contributions

CAE and AM conducted BAC library screening and probe characterisation, WR and OC conducted FISH experiments, and TH conducted gene characterisation. CAE and WR carried out *in silico *analysis. CAE, WR, AFS drafted the manuscript, ID, MAFS and JMG contributed reagents and provided input to the manuscript, AFS and MAFS conceived, designed and coordinated the study.

## Supplementary Material

Additional file 1 Probes used for BAC library screening, shows the number of BACs identified by each probe. Click here for file
