# Supplementary material for: The evolution of imprinting: chromosomal mapping of orthologues of mammalian imprinted domains in monotreme and marsupial mammals
Source: BMC Evol Biol. 2007 Sep 6;7:157. doi: 10.1186/1471-2148-7-157 (PMC2042987; doi:10.1186/1471-2148-7-157)
Supplement: Additional file 1 — Probes used for BAC library screening, shows the number of BACs identified by each probe. [file 1471-2148-7-157-S1.doc]

| **Locus** | **Genbank Sequences used for probe design** | **Forward Primer** | **Reverse Primer** | **Size** | **Annealing T** | **Platypus BACs identified** | **Wallaby BACs identified** |
| --- | --- | --- | --- | --- | --- | --- | --- |
|  |  |  |  |  |  |  |  |
| IGF2 | AF225876 | 5’-GGCATCGTGGAGGAGTGTTGC | 5’-CTCGGACTTGGCAGGGGT | 81bp | 60^o^C | 2 | 10 |
|  |  |  |  |  |  |  |  |
| IGF2R (platypus) | AF151172 | 5’-GGACACATCACCACCAAGTG | 5’-CATTCCTTCTGGCCATTGAG | 224bp | 55^o^C (40 cycles) | 10 |  |
| IGF2R (wallaby) | AF339159 | 5'-GCACAGTTCCTAAGCTTACTGC | 5'-TTTGGGTGAGAGGGTTAAGG | 156bp | 55^o^C |  | 8 |
|  |  |  |  |  |  |  |  |
| DIO3 | NM_001362 AF426023 U24282 XM_426465 | 5’-GCAGCTGTACCTGACCACCG | 5’-GACCCAGCCGTCGGATGGGTG | 128bp | 60^o^C | 15 | 6 |
|  |  |  |  |  |  |  |  |
| GRB10 | XM_376609 NM_010345 | 5’-GTCTTTAGTGAAGATGGGAC | 5’-GTCCAGCTGTTGTCATCCAC | 116bp | 55^o^C | 19 | 15 |
|  |  |  |  |  |  |  |  |
| GNAS | NM_000516 BC080816 NM_019132 XM_417485 | 5’-TCATCTTCGTGGTGGCCAGC | 5’-GTTCCAGATGCTCTTGAAGAG | 104bp | 50^o^C (40 cycles) | 9 | 18 |
|  |  |  |  |  |  |  |  |
| UBE3A | NM_000462 NM_011668 XM_416882 | 5’-GCGAGCAGCTGCAAAGCATC | 5’-CCTTTCTTGGAGGGATGAGG | 198bp | 60^o^C | 7 |  |
|  |  |  |  |  |  |  |  |
| SLC38A4 | NM_018018 | 5’-GTGATTTACAAGAAATTCCA | 5’-CGGGAGTTGAATACAAAGTA | 275bp | 55^o^C | 10 |  |
|  |  |  |  |  |  |  |  |
| DLK1 (Wallaby) | TI_395847291 | 5'-CTTGTTGAAGAAAACAATGC | CCAGGCCATCTGCTTCACCA | 81bp | 55^o^C |  | 13 |
| DLK1 (Platypus) | TI_752207707 | 5'-ACAGGGCCCTTTCCTAAGAT | 5'-ACTCACCCTTGCTCTGCAAC | 129bp | 55^o^C | 15 |  |
|  |  |  |  |  |  |  |  |
| MRPL23 | NM_021134 | 5'-GTACCCCCTGTACCGGCTGGG | CCATGGGGATCCGGAACTGCAC | 122bp | 60^o^C |  | 19 |
| CD81 | NM_004356 | 5'-CATGGGAGTGGAGGGCT | 5'-GTCCGGGTCACTTAGGGG | 767bp |  |  | 5 |
|  |  |  |  |  |  |  |  |

**Table S1 – Sequences and PCR primers used to generate probes for each gene studied.**

For DIO3, GRB10, GNAS and UBE3A primers were designed to the well conserved regions in the mouse sequence. All other probes were designed to the sequence in column 2. The number of Platypus and Wallaby BACs identified are shown in columns 7 and 8.
